# Notes

**Published:** 1902-10

**Authors:** 


### NOTES.

### SCORE CARD.

Date ....................

Owner's { Name ....................
Address ....................

Specimen's { Reg. No. .................... Ear Tag No. ....................

Dropped ....................

| *General Classification.* | *Score Points and Count.* | *Possible Count.* | *Cuts on Points.* |
|---|---|---|---|
| Head . . 4 | Head small and lean | 1. | |
| | Face dished | 0.5 | |
| | Broad between the eyes | 0.4 | |
| | Narrow between the horns | 0.2 | |
| | Horns small and well formed | 0.2 | |
| | Eyes full, prominent, and placid | 0.2 | |
| | Jaw strong | 1 | |
| | Muzzle large and broad | 0.3 | |
| | Nostrils large and open | 0.2 | |
| Neck . . 6 | Neck thin | 2 | |
| | Neck rather long | 2 | |
| | Neck with clean-cut throat | 0.5 | |
| | Neck not heavy at the shoulders | 1 | |
| | Neck thin at withers | 0 5 | |
| Body . . 23 | Back level to the setting on of tail | 5 | |
| | Broad across loins and straight to hips | 3 | |
| | Barrel deep through heart and flank | 5 | |
| | Body wedge-shaped and long | 5 | |
| | Body with deep, large paunch, well hooped | 5 | |
| Legs. . . 2 | Short legs well formed | 2 | |
| Tail . . . 2 | Tail well set on | 0.5 | |
| | Tail thin | 0.5 | |
| | Tail long | 0.5 | |
| | Tail with good switch | 0.5 | |
| Hips . . 10 | Hip bones wide apart | 3 | |
| | Hip bones high and prominent | 3 | |
| | Rump long | 2 | |
| | Thighs long, flat, and well cut out | 2 | |
| Udder . . 27 | Udder large, well formed, and not fleshy | 5 | |
| | Fore udder full in form and well rounded | 5 | |
| | Fore udder well up on belly | 5 | |
| | Rear udder full in form and well rounded | 5 | |
| | Rear udder well out behind | 2 | |
| | Rear udder well up behind | 3 | |
| | Quarters must be well formed together and not cut up between teats | 2 | |
| Teats . . 6 | Teats rather large, but not over size or small | 2 | |
| | Teats wide apart | 2 | |
| | Teats squarely placed | 2 | |
| Milk veins 5 | Milk veins large, prominent, and elastic | 5 | |
| General . 15 | General dairy conformation | 5 | |
| | General appearance | 2 | |
| | General apparent constitution | 2 | |
| | General apparent capacity | 2 | |
| | General condition of hide | 2 | |
| | Disposition quiet | 2 | |
| Total, 100 | Possible score | 100 | |
| | Total cuts | | |
| | Net score | | |

Scorer ....................

—*Jersey Bulletin*, October 15, 1902.

